# A critical review of the US Senate examination report on the 2021 US Capitol riot

**DOI:** 10.3389/fpsyg.2023.1038612

**Published:** 2023-02-01

**Authors:** Hakkyong Kim, Julak Lee

**Affiliations:** ^1^Department of Convergence Security Engineering, Sungshin Women’s University, Seoul, Republic of Korea; ^2^Department of Industrial Security, Chung-Ang University, Seoul, Republic of Korea

**Keywords:** US Capitol riot, legitimacy and power, procedural justice, dialogic approach, negotiated management, dialogue police

## Abstract

The US Capitol riot on January 6, 2021, is currently regarded as an unprecedented armed occupation of the US Capitol that left an irreparable stain on the history of American democracy. Five months later, as part of reflective practice, the US Senate released an examination (review) report on the response failures of federal and local law enforcement agencies, along with corresponding recommendations. This research seeks to critically analyze not only response failures but also recommendations made by the US Senate with a comprehensive theoretical framework incorporating concepts of (1) legitimacy and power, (2) procedural justice, and (3) crowd psychology and the protest policing model. In the end, the research tries to present a practical cooperative dialogue or at least a dialogic approach as a means of strengthening legitimacy based on procedural justice, given that not only European countries but also the Republic of Korea currently operate ‘(Korean and Swedish) Dialogue Police’ and (British) ‘Police Liaison Officer’ in practice.

## Introduction

The First Amendment to the United States Constitution comprehensively guarantees the freedom of religion, freedom of speech, freedom of the press, freedom of peaceful assembly, and the freedom to petition. The US Capitol Riot that broke out on January 6, 2021, also began with a seemingly peaceful protest based on the First Amendment to the Constitution mentioned above. However, what had begun as a peaceful protest quickly escalated into an armed occupation of the Capitol, unprecedented in the history of American democracy, resulting in four deaths and hundreds of injuries. On June 8, 2021, the US Senate examination report, which examined the issues with the response of federal law enforcement agencies and the United States Capitol Police (USCP) and made recommendations for improvement regarding the Capitol riot, was finally released.

In the US Senate examination report mentioned above, only four issues were listed as problems with the response of the USCP, which has jurisdiction over the Capitol: (1) lack of riot control training and equipment, (2) absence of a comprehensive response plan at an organizational level, (3) negligence of riot control equipment management, (4) issues with the analysis and distribution of information about the risks (only being shared within the organization). Furthermore, the recommendations for improvement are solely focused on reinforcing the ability to respond reactively, such as (1) establishing a permanent Civil Disturbance Unit (similar to the South Korean riot police), (2) reinforcing both basic and advanced riot control training, and (3) preparing a holistic response and police force deployment plan (US Senate, 2021).

Professor Clifford Stott, a British expert on crowd psychology, explains the mechanism by which protests degenerate into riots using the theories of Legitimacy and Power (Stott, 2009; Reicher and Stott, 2020). Examining the contents of the Senate report with this theoretical framework, it can be argued that the problems with response and recommendations for improvement presented in the Senate report are drawn solely from the perspective of *Power*. Nevertheless, no prior studies have critically analyzed the power-based recommendations for improvement.

Taking these problems and limitations into account, this study aims to examine the overview of the US Capitol riot incident, the issues with the response (of related organizations), and the recommendations for improvement (on such problems) based on the contents of the US Senate report and analyze them with the theoretical framework of Legitimacy and Power. Furthermore, beyond the psychological change mechanism of Legitimacy and Power, this study attempts to comprehensively apply the theory of Procedural Justice from criminal policy, the Elaborated Social Identity Model from crowd psychology, and Negotiated Management from protest control models as a framework of critical analysis. In this study, the documentary analysis research methodology was used mostly to achieve the aforementioned research purpose. This is because documents can capture even the smallest information about systems, policies, and events beyond our comprehension, as we cannot directly experience them each in detail. In this context, document analysis is often referred to as a key methodology that allows the researcher to dig deep down to the truth of the systems, policies, and events. First, in this study, we attempted to thoroughly analyze the US Senate report (mentioned above) as a document for documentary analysis. Second, the protocols of Sweden’s Dialog Polis, the UK’s Police Liaison Officers, and Korean dialogue police were examined through documentary analysis based on Legitimacy (not ‘Power’), Procedural Justice, the ESIM theory, and Negotiated Management. Finally, we attempted to present these protocols as a supplementary alternative based on ‘Legitimacy’ (not ‘Power’) to the US Senate report’s conclusion. Official documents or reports were used to analyze the specific protocols of each organization. Below, the framework of analysis used in this study will be examined as a theoretical background.

## Theoretical background – Framework of analysis

**Figure 1:**
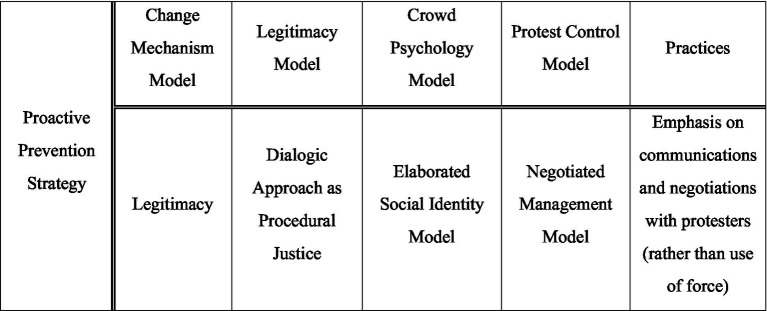
Theoretical background – framework of analysis.

As will be explained afterwards, Sweden, the UK, and South Korea have a dedicated unit that alleviates conflict through dialogue and negotiation with the protest organizer before the protest takes place. Such dedicated units adopt the Elaborated Social Identity Model as a crowd psychology theory and the Negotiated Management model as a protest control model. However, the means that such dedicated units utilize, dialogue and negotiation, can also be linked to the Legitimacy theory and the Procedural Justice theory. Therefore, in this study, we will examine the contents of Legitimacy, Procedural Justice through Dialogic Approach, the ESIM Theory, and the Negotiated Management theory as the framework of analysis (Figure 1).

### Legitimacy and power

Professor Clifford Stott presents two concepts, legitimacy and power, regarding the mechanism through which peacefully initiated protests degenerate into radical and unlawful violent riots. This mechanism, referred to as the Change Mechanism (of crowd psychology), is characterized by the breakdown of the typical crowd psychology into disorder and chaos (Stott, 2009, 2011).

Assuming that the crowd violates the law during a protest, the police have no choice but to perceive their actions as illegitimate, as they violate the current law. From the crowd’s point of view, however, their actions (regardless of whether they violate the law) are bound to be perceived as legitimate (necessary to achieve their goals). In such instances of legitimacy conflict, the mechanism that regulates the shift in crowd psychology leads to a problem of power. However, in the early stages, the police inevitably have a deterrent advantage in terms of power due to their trained police forces, sufficient resources such as riot suits and riot control gear, and particularly the legal authorization for the use of physical force and forceful dispersal.

However, the police’s indiscriminate or excessive use of physical force against a violation of the law will cause the crowd to perceive such intervention as ‘Illegitimate’, thus transforming the crowd into an ‘Entity’ with a united identity to fight against the police. The problem, however, is that in large-scale protests and events, the crowd usually outnumbers the police. For instance, assume the crowd comprises 1,000 people, and 200 riot police officers are mobilized. At the beginning of the protest, the crowd is not composed of a single group but of individual crowd groups with multiple social identities. In other words, although there are a total of 1,000 people, not all of them initially perceive each other as the same crowd with a single social identity. The 1,000 people are a gathering (each with different social identities) of separate individual groups of 50–100. At this juncture, it is apparent that the well-armed and highly-trained 200 riot police officers are superior in ‘Power’ to a crowd of 1,000 people composed of individual groups with diverse identities.

In this case, if the crowd psychology transforms into a single entity with a unified identity due to factors such as the police’s excessive use of physical force, the crowd gains ‘Power’. This phenomenon is called ‘Empowering’, and causes a psychological conflict of ‘Us vs. Them’ because of the perception of the police’s reaction as being illegitimate (Stott, 2009, 2011; Cheung, 2021). Under this structure, the police are outmatched in terms of power when confronting a crowd with a united social identity. Of course, in a situation that the police fall behind the crowd in a power conflict, they may reinforce deterrence by employing additional police forces or higher levels of physical force to reach a position of power superiority. The crowd, however, will again stand up against the police by raising the level of violence and unlawful activities, and if this happens, the power conflict between the two sides will eventually result in a vicious cycle of violence and confrontation (Vitale, 2005; Vitale et al., 2011).

According to Stott et al. (2012), the proactive prevention of chaos and disorder is significantly more important than a reactive response to the occurrence of extreme chaos and disorder. Furthermore, they emphasize that communication and negotiation may be the most effective methods in terms of the proactive prevention model. Furthermore, they argue that communication and negotiation have a positive impact on the (crowd’s) perception of the ‘Legitimacy’ of police action and that these means are crucial in preventing an ordinary protest crowd psychology from turning into a radical, illegal, and violent single-identity crowd psychology.

### Procedural justice theory and dialogic approach theory

As previously mentioned, Stott et al. (2012) emphasize communication and negotiation as a proactive model for preventing chaos and disorder. Here, the question “When the crowd and the police have different perceptions of legitimacy, on what theoretical basis can communication and negotiation narrow the gap in the perception of legitimacy?” may be posed. One of the theoretical answers to this question is the Procedural Justice (PJ) theory on police legitimacy.

According to Tyler (2006), one of the leading scholars on police legitimacy, citizens comply with the law not out of fear of punishment but because they respect the legitimate authority of the government (such as the police) or the system that enforces the law. Furthermore, many previous studies on police legitimacy, including by Tyler (2006), empirically demonstrate a correlation between the perception of procedural justice (PJ) and increased police legitimacy, resulting in increased cooperation with the police (e.g., Sunshine and Tyler, 2003; Tyler and Fagan, 2006; Hough et al., 2010). However, the existing studies on legitimacy based on procedural justice have limitations in that the discussions were conducted primarily from the Audience Legitimacy (e.g., Beetham, 1991; Coicaud, 2009).

Recognizing these limitations, many studies have attempted to analyze the legitimacy based on procedural justice not only from the perspective of citizens but also at the level of power holder legitimacy, i.e., the legitimacy of the police who carry out the actions (e.g., Jacinta and Rod, 2010; Bottoms and Tankbe, 2012; Mazerolle et al., 2013; Martin and Bradford, 2021). Since then, the assertion that a dialogic approach (DA) is one of the preventative measures the police should employ to ensure legitimacy has gained persuasive power. The findings of these studies indicate that dialogue, or in other words, a ‘Dialogic Approach (DA)’ between citizens and the police (concerning the police’s act of disposition), is the essence of justification procedural justice (Jacinta and Rod, 2010).

To summarize, the police’s active effort to communicate, or the dialogue process, forms one of the components of legitimacy based on procedural justice. Such efforts at initiating dialogue are a means of narrowing the gap *vis-à-vis* perceptions of legitimacy between the police and citizens. Furthermore, engaging in the dialogue can simultaneously improve both the legitimacy based on procedural justice and the ‘Cooperation with the Police.’ Ironically, even if such dialogue efforts do not narrow the gap in the perception of legitimacy, the police’s active and sincere effort to communicate or the dialogue process itself is an important means of attaining legitimacy. Due to the fact that the theory of legitimacy based on procedural justice itself is already being applied at protest sites (Maguire, 2015; Tyler et al., 2018; Snipes et al., 2019), the police’s dialogic approach is another important means to ensure legitimacy based on procedural justice.

### Elaborated social identity model and negotiated management theory

Among protest control methods, theories such as legitimacy, procedural justice, and dialogic approach can be regarded as being (relatively) close to the proactive prevention model. Such a preventive model is also connected to the Elaborated Social Identity Model (ESIM) theory (a protest crowd psychology model) and the negotiation management theory (as a protest control method; Stott et al., 2012; Radburn et al., 2018). In contrast, the use of force based on power corresponds to a reactive response model, which is associated with the Classic Crowd Psychology (CCP) theory. This was developed as a protest control method in the late 19th century by a French scholar named ‘Gustave Le Bon and is associated with the escalated force (EF) model (Stott, 2011).

In contrast, the use of force based on ‘Power’ corresponds to a ‘Reactive Response’ strategy, which is linked to the Classic Crowd Psychology (CCP) theory, which was developed by a French scholar named Gustave Le Bon in the late 19th century, and is connected to the Escalated Force (EF) model as a protest control method. However, since none of the advanced nations employ the ‘Escalated Force’ model in its purest form, most of the current protest control methods based on ‘Power’ should be viewed as ‘Command and Control (CC)’ or ‘Strategic Incapacitation’ (Vitale, 2005; Gillham, 2011; Vitale et al., 2011).

The Classic Crowd Psychology (CCP) theory views the crowd as an irrational group with a single identity. It holds that crime or deviant behavior occurs naturally as a result of individuals losing self-control upon entering the crowd due to anonymity. It is predicated, in particular, on the premise that if a minor illegal activity is tolerated, a contagion of illegality and violence would readily spread throughout the crowd due to anonymity. For this reason, when a minor illegal activity occurs, the Escalated Force (EF) model (based on the Classic Crowd Psychology (CCP) theory), the Command and Control (CC) model (partially), or the Strategic Incapacitation (SI) model focus on quickly preventing the spread of such activities through the immediate use of force (Vitale, 2005; Gillham, 2011; Vitale et al., 2011).

The ESIM theory, on the other hand, regards the crowd as a heterogeneous group with diverse identities capable of rational judgment, communication, and self-policing (Reicher et al., 2004; Her Majesty’s Chief Inspector of Constabulary [HMCIC], 2009). External stimuli or responses, notably police responses, are considered significant influencing factors as several groups of individuals with different tendencies merge into a (single) crowd of people with a single social identity. If the police use physical force in a way that is neither immediate nor proportional to minor illegality or violence committed by some groups of the crowd, the social identity of the entire crowd unifies and forms a structure in which it fights against the police. Therefore, the ESIM theory places emphasis on promoting self-policing through means such as negotiation, dialogue, communication, and tolerance of minor illegalities.

Therefore, the negotiated management (NM) model based on the crowd psychology of the ESIM theory emphasizes dialogue and negotiation with the organizers and participants to minimize unnecessary stimulation and confrontation, with the use of force being the last resort in accordance with the principle of subsidiarity (Porta and Reiter, 2006). Furthermore, the group that commits illegality is differentiated from the rest of the peaceful crowd, and the use of force is restricted to the former.

## Content of investigation of the US Senate report on the US Capitol riot

### Problem in response

On June 8, 2021, the US Senate published an examination report the failures in response to the US Capitol occupation (riot) on January 6, 2021. The report presented the following response problems of the federal law enforcement agencies, including the FBI and the USCP.

### Problems of collecting, analyzing, and distributing threat information

#### Federal law enforcement agency level

The Department of Homeland Security (DHS) is the primary federal law enforcement agency in the United States that collects and evaluates protest-related threats. However, none of the agencies had issued or a formal threat assessment or intelligence bulletin regarding the possibility of a Capitol occupation on January 6. These federal agencies presumed that a simple and typical protest would be held in accordance with the First Amendment, which guarantees the freedom of peaceful assembly. In this regard, Steven Sund, the director of the USCP at the time, testified that the response failure was primarily due to the failure of federal law enforcement agencies to provide accurate information. In particular, he testified that he had an emergency meeting with the FBI, Secret Service, DCNG, and others the day before the occupation; yet no mention was made of the possibility of the protests degenerating into riots at these meetings.

#### United States capitol police level

According to the Senate report, the Intelligence and Interagency Coordination Division (IICD) of the USCP had already collected several social media posts inciting illegalities and violence, including Capitol intrusion, at a protest scheduled for January 6. Furthermore, it has been confirmed that they received multiple anonymous reports prior to January 6, indicating that protesters would break into the Capitol and that far-right groups such as the Proud Boys would participate in the January 6 protest. They had already compiled a report stating that the likelihood of illegal violence would increase if these far-right groups were to participate in the protest.

Nevertheless, the Daily Intelligence Report issued by the IICD regarding the January 6 protest did not accurately reflect the previous report on the Proud Boys. As a result, the probability range of specific risks, such as civil disturbances and riots, was predicted to be, remote (low probability of occurrence) or improbable at most. To put it another way, the USCP did not thoroughly assess or process its own intelligence.

### Lack of comprehensive response plans and riot control training, and problems with the provided equipment

#### Lack of comprehensive response plans at a United States capitol police level

The Senate report also notes that the USCP was unprepared for a comprehensive response to the probability of violent riots during the January 6 protest. Particularly, there were no department-wide plans for response or police force deployments, only security measures for individual departments, such as USB and the Civil Disturbance Unit (CDU), a temporary riot response unit. However, given that these documents were only one or two pages long, they could not be deemed effective security measures.

#### Lack of riot control training and equipment

It was also confirmed that the on-site police officers mobilized in response to the protest did not receive proper riot control training and were not adequately equipped with equipment such as riot suits. Moreover, only seven platoons of ‘Civil Disturbances Units’ (CDU) were initially mobilized on January 6, with a total of 160 officers; however, only four of these platoons were equipped with riot suits and shields. The remaining three were pre-deployed (without protective clothing or riot control equipment) and, despite being members of the CDU, they had only received basic riot control training rather than advanced riot control training. As the situation deteriorated, a total of 1,200 police officers were mobilized; however, only 300 had received proper riot control training and protective equipment, while the remaining 900 had received no protective equipment, let alone any proper riot control training since their recruit training.

#### Negligent management of riot control equipment

The negligent management of riot control equipment was mentioned as another issue. For example, many shields were shattered on the first impact since the riot shields supplied to the police officers mobilized on January 6 had not been maintained for a long time. Furthermore, despite the fact that the protests were degenerating into riots, only equipment such as the rubber ball gun (FN-303) and pepper balls were allowed, while the use of less-lethal weapons such as sting ball grenades and grenade launchers that are effective for crowd control was prohibited.

### Problems of delay in requesting DCNG mobilization and dispatch

The US Senate report confirms that despite the worsening of the situation on January 6, both the request for the DCNG and its actual dispatch were delayed for a considerable period of time.

#### Delay in approval of the request for the District of Columbia National Guard (DCNG)

As was stated earlier, for the USCP to receive assistance from other institutions, such as the DCNG, the director must first request a written approval from the Capitol Police Board (CPB). The CPB must then declare a state of emergency before approving requests for DCNG mobilization. On January 6, as the situation worsened, Steven Sund, the director of the USCP at the time, submitted a written request to the CPB for approval of DCNG mobilization. However, the CPB, which has the authority to approve, disagreed over whether to base its approval on a majority or unanimity, causing a delay in the approval itself.

#### Delay in DCNG dispatch

Since the Black Lives Matter (BLM) protests of 2020, the DCNG’s riot control procedures have become more stringent. These are: (1) The DCNG must obtain prior approval from the US Secretary of Defense in advance to use weapons, police rods, or protective equipment. (2) The Quick Reaction Force (QRF) should only be mobilized as a last resort. (3) A concept of operation must be prepared before mobilizing the DCNG, and the US Secretary of the Army must approve the concept of operation for the QRF to be mobilized.

### Recommendations for improving the problems

This section will review the general recommendations for improvement made in the US Senate examination report regarding the identified response issues.

#### Capitol police board

The following recommendations were made to the CPB: (1) Consider revising the relevant laws so that the head of the CPB may request the *ex officio* mobilization of the DCNG to the US Army in the event of an emergency. (2) Ensure that the board members regularly review the various policies and procedures of the USCP to fully understand and become familiar with their contents.

#### United States capitol police

First and foremost, the Senate report states that all USCP officers must receive basic riot control training annually. It also recommends providing them with the most up-to-date riot control equipment and routine management. Second, a department-wide plan for response and police force deployment should be prepared. These plans are recommended to include a comprehensive list of threat assessments for the event, police deployment strategies, operational objectives, incident command systems, authorized levels of the use of force, and related emergency plans. Third, it is proposed to establish a permanent Civil Disturbance Unit (CDU) with dedicated police officers receiving annual advanced riot control training and equipped with professional riot control equipment. Finally, it is proposed to establish a new Intelligence Bureau (IB) to integrate the USCP’s decentralized information collection and analysis functions and provide police officials with regular and professional training in information collection and analysis.

#### Federal law enforcement agencies

The following are the recommendations for federal law enforcement agencies, including the FBI: (1) Re-examine and evaluate the process of analyzing and processing public information (such as social media content containing violent threats). (2) Re-examine and evaluate the standards for issuing and distributing information reports to consumer agencies, such as the USCP. (3) Comply with statutory reporting requirements for Congress regarding domestic terrorism-related information, such as threat levels.

#### DC national guard

Finally, the following recommendations are made for the DCNG: (1) prepare (in advance) a concept of operation for riots and terrorist incidents to enable immediate mobilization. (2) Conduct training on the mobilization of National Guard units from other jurisdictions to provide immediate assistance in the event of an emergency. (3) In the event of an emergency in the Capitol, Proximity and Response time should be given top priority when deciding where to deploy the QRF. (4) Establish a clearer final approval procedure for DCNG mobilization to avoid approval-related mobilization delays.

## Criticism and alternatives to US Senate recommendations for improvement

### The problem of focusing only on power and a suggestion for improvement

As stated previously, the mechanisms of change in crowd psychology can be categorized broadly into legitimacy and power. First, legitimacy, a mechanism of change, is associated with procedural justice, and procedural justice is associated with a dialogic approach. It is related to the ESIM theory as a crowd psychology model and to the negotiated management model as a method of controlling protests. Consequently, all theories pertaining to legitimacy fall into the category of proactive prevention. However, power, another mechanism of change, is based on the deterrence theory, which emphasizes using physical force. Such suppressive concepts are associated with the Classic Crowd Psychology (CCP) theory (as a crowd psychology theory) and the Command and Control (CC) theory or the Strategic Incapacitation (SI) model (as a protest control method). Therefore, the theories on power appear to be generally consistent with the reactive response (suppression) model.

The proactive prevention model, which strengthens legitimacy, should therefore serve as a guiding principle of protest control methods. However, in exceptional cases where legitimacy-strengthening tactics fail or the time to deploy legitimacy tactics is limited due to immediate occurrence of specific threats, power-based reactive response tactics (including the use of physical force) may be used as pre-emptive measures. To put it another way, when discussing the police’s approach to protest, power is essential for maintaining public peace and order, but legitimacy is just as important as power for protecting constitutional rights. In other words, a balanced approach to preparation is essential.

All of the problems regarding response presented in the US Senate report stem solely from the perspective of power. As a result, all recommendations for improvement focused solely on improving strategies or tactics based on power. The issue is that no content or analysis regarding strengthening legitimacy as a proactive prevention model was suggested. Such facts demonstrate how the ‘command and control’ model and the ‘classic crowd psychology’ theory based on ‘physical force’ are deeply ingrained in the inherent considerations of US law enforcement agencies (including the US Senate).

Furthermore, as a problem associated with ‘Crowd Psychology’ it can be criticized that the report does not contain an analysis of the process of change in crowd psychology. The Senate Report, which relies solely on ‘Power’, appears to presuppose the intrinsic crowd psychology (based on CCP) as a violent social identity already unified with the intention of occupying the US Capitol from the beginning. However, based on this premise, it is difficult to explain the relatively peaceful crowd psychology of the protest from January 5 to the morning of January 6. In contrast, according to the ‘ESIM Theory’, it can be interpreted that at some point, the subgroups with diverse individual social identities present at the rally on January 6 evolved into a crowd psychology with a violent social identity (especially due to external factors).

If this analysis is to be valid, the US Capitol Police missed the opportunity for a differentiated response (to the former) by separating groups instigating unlawful violence (riot) from groups that had gathered to support President Trump (without the intention of rioting) before the social identity of the crowd was unified. Eventually, such responses that only consider “Power” (especially if the ESIM Theory is applied) lead to overreactions to minor infractions or disturbances, which can lead to adversarial tension of “Us vs. Them” between the police and the protesters. As a result, there could be a continual cycle of violence and physical conflict.

Numerous prior studies have criticized the propensity of US law enforcement agencies to prefer power-based strategies or tactics. Maguire and Oakley (2020) and Maguire et al. (2021) specifically point out that the US police’s approach to handling protests relies heavily on tactical methods to suppress riots and neglects the more comprehensive strategies required to prevent conflicts or violence. In light of this, the studies of Maguire and Oakley (2020) and Maguire et al. (2021) emphasize that protest response training of US law enforcement agencies should place a greater emphasis on prevention and de-escalation strategies to minimize the use of physical and deterrent force.

### Complementary alternatives – Strengthening legitimacy through dialogue and negotiation

The UK, Sweden, and South Korea have established and are operating a dedicated unit (as a separate unit from the Riot Police) that alleviates conflict through dialogue and negotiation by contacting the protest organizers (before the protest). We propose this system as an alternative (in terms of establishing Legitimacy) to the protest control method of the US police agencies (which relies only on Power). Below, we will take a closer look at the police units in each country that alleviate conflict on the protest sites and the specific protocols of these units.

#### 1.1.1. Sweden – Dialog Polis

In 2001, when the EU summit was held in Gothenburg, Sweden, over 50,000 anti-globalization protesters from all over Europe, including Sweden, gathered to protest against the summit’s agenda, internationalization, and globalization. This protest, however, degenerated into violent clashes with the police, resulting in the arrest of 575 protesters and the wounding of approximately 400 police officers. Three more were wounded as a result of the police’s use of firearms. These riots remain in the memory of the Swedish people as a national trauma. In 2004, the Swedish National Police Board established Special Police Tactics (SPT) based on the ideas of Dialogue, De-escalation, and Non-confrontation (Swedish National Police Board, 2010; Holgersson and Knutsson, 2011; Knutsson, 2017). The Dialog Polis is the key unit of the SPT, and their roles and responsibilities are based on five principles, (a) Negotiation, (b) Mediation, (c) Suggesting, (d) Communication, and (e) Monitoring. The activities of the Dialog Polis can be divided into 3 stages in chronological order, and are as follows: (a) Activities before a protest, (b) Activities in the protest site, and (c) Activities after a protest. One of the important attitudes that Dialog Polis officers must equip in these activities is “seeing the police action from the protester’s point of view.” The specific activities of Swedish Dialog Polis unit are as follows.

##### 1.1.1.1. Before a protest

First, the police must attempt to build trust by reducing irritation in the messages of their media statements. This is because a negative prejudice against the protest organizers in the police’s media statement can lower the possibility of a peaceful protest. In addition, if the protest contains a considerable risk of conflict, it is necessary to coordinate the time and site of the protest, whether a march is planned and the march route if so. At the same time, the requirements of the protesters must be negotiated with. Furthermore, they must intervene to ensure that the tactical commander of the security operation and the protest organizers can exchange information. They must also provide the tactical commander of the security operation with a number of probable scenarios based on the situation or police action. Finally, for smooth coordination and negotiation, involving “external actors” such as civic organizations, religious groups, and volunteer groups must also be considered.

##### 1.1.1.2. During a protest

At this stage, the main task of the Dialog Polis is to act as a link between the organizers or protesters and the tactical commander of the security operation. Even if no meaningful dialogue is made, the presence of the Dialog Polis wearing a vest on-site, plays a significant role itself.

Particularly, wearing plain clothes with a ‘Dialog Polis’ vest on-site while performing activities publicly reduces the police’s anonymity and has the effect of moderating the protesters’ behavior. Due to this public activity, Swedish Dialog Polis officers can observe the progress of the protest from the protesters’ point of view (and not the police), and assess the actions of the public security police more objectively.

The Dialog Polis officer should ensure that the results of negotiation with the protest organizers before the protest are being kept on-site, and if necessary, should make a new negotiation through dialogue. During the protest, they must identify the police’s response and the change in protester atmosphere (such as changes in crowd psychology) responding to it, and if necessary, provide the tactical commander of the security operation with a way to minimize violence and additional information about it. If the external actors are at the protest site, it is necessary to provide them with the relevant information as well. The Dialog Polis are responsible for coordinating the activities of these groups as well.

##### 1.1.1.3. After a protest

It is also important to hold a follow-up meeting with the protesters for “feedback” on the police operation or the Dialog Polis action. An explanation to the protest organizers about the police actions that occurred during protest may also be required if necessary. On the other hand, it is also necessary to receive feedback on the protesters’ behavior from the public security police. Finally, after the protest is over, a “Debriefing” should be held between the police officers involved in the protest operation.

#### 1.1.2. UK – Police Liaison Officers

Currently, in the UK, protest-related communication or conflict management is handled by Police Liaison Officers (PLO) (Her Majesty’s Chief Inspector of Constabulary [HMCIC], 2009; College of Policing, 2015). In addition to conflict management, PLOs provide security commanders with information such as the atmosphere of the protest or changes in crowd psychology. Similar to the Swedish Dialogue Police, the PLO system is considered to promote peaceful protests while reducing on-site disturbance. The UK’s Police Liaison Officer’s activities and responsibilities are similar to that of the Swedish Dialog Polis, based on the five principles: (1) “Negotiation,” (2) “Mediation,” (3) “Initiation,” (4) “Communication,” and (5) “Sensing.” We intend to analyze the activities and responsibilities of the Police Liaison Officers divided into 3 stages in chronological order: (a) Before a protest, (b) During a protest, and (c) After a protest. The specific activities of these Police Liaison Officers are as follows.

##### 1.1.2.1. Before a protest

Before the protest, it is strongly encouraged that the Police Liaison Officers hold a pre-event engagement with the protest organizer. This is because such pre-event engagement helps build a relationship between the two entities. From the protester’s point of view, they can learn the point of contact with the police and how the police will respond to their requirements. Such pre-event engagement can increase communication with the police, and in particular, can inform the police on precisely what they want through their protest and gain the police’s cooperation in this regard. On the other hand, when mobilized to a protest site without any pre-event engagement or meeting, there is a chance that the Police Liaison Officer’s presence could be perceived as a “hindrance” to the protest organizers.

The police can also know what the protesters will be doing at the protest in advance and they can dialogue with the protest organizers about “unacceptable behavior” based on this. This method is known as the “No surprises approach.” Also, from a police tactical commander’s point of view, it is advantageous in that it can lead to a more efficient distribution of the input resources based on the contents of negotiation acquired through dialogue and discussion.

##### 1.1.2.2. During a protest

Police Liaison Officers should be deployed mainly near the protest site, and the riot control police force should be deployed at some distance from the site so that the riot control police force may focus only on the ‘consequential’ situations. Especially from a tactical commander’s point of view, since “the perception made by observing the crowd from the inside can be quite different from that of the outside,” a Police Liaison Officers may provide more accurate information on the crowd’s psychology or the reciprocal relations. On the other hand, by communicating with the on-site Police Liaison Officers rather than with several police officers, the protester can connect with the entire police force and receive feedback on the overall situation.

##### 1.1.2.3. After a protest

Since the “end” of one protest becomes the ‘beginning’ of another, maintaining a relationship with the protest organizer is crucial even after a protest. To do so, it is necessary that the police and the protest organizers exchange feedback on each other’s actions. Local traders in the vicinity of the protest site also prefer feedback from these protest organizers since they want to check whether their actions may trigger protesters, and avoid doing it if so.

#### 1.1.3. South Korea – Korean dialogue police

Korea has also been running a system known as the Korean dialogue police since October 2018, modeled after the Swedish Dialogue Police system. After undergoing professional training, the police officers (from the police intelligence department) who have been managing protests on-site (in civilian clothes) now work as dialogue police officers. Unlike before, they work in public and wear uniforms marked ‘Dialogue Police’. Like their foreign counterparts, their role is to improve communication with protest organizers and participants to alleviate conflicts and promote peaceful protests (McPhail, 1997; Jeong, 2020; Korean National Police Agency [KNPA], 2020a). Another role of the Korean dialogue police is to stay either close to or within the crowd, accurately detect shifts in crowd psychology, and alert the riot police of the shift.

The five main tasks performed by the Korean dialogue police are as follows: (a) negotiation, (b) mediation, (c) communication, (d) monitoring (gathering public information, not secret criminal intelligence), and (e) suggesting practical alternative resolutions. The execution of these five tasks is a manifestation of the role that the dialogue police play in minimizing the risk of potential conflicts and confrontations at protest sites and ensuring peaceful and secure public gatherings. Like the Swedish Dialog Polis and the UK’s Police Liaison Officers, its work can be distinguished into three stages: (a) before a protest (Proactive), (b) during a protest (Active), and (c) after a protest (Reactive; Korean National Police Agency [KNPA], 2020b; Ministry of the Interior and Safety (in Korea), 2020; Kim, 2022).

##### 1.1.3.1. Before a protest (Proactive)

In order to organize a protest or march in Korea, the organizer is required by law to submit a written notice or report to the local police station with jurisdiction over the protest site or march route. Before the protest, a senior Korean dialogue police officer with authority contacts the organizer(s) and tries to establish rapport. If necessary, the details, such as protest locations and march routes, etc., can be discussed with the protest organizers to keep them informed of potential police actions. To minimize complaints and conflicts, they work on a compromise or an acceptable alternative to potentially problematic plans until the senior Korean dialogue police officer agrees to the specific requirements of protest organizers. Some senior Korean dialogue police officers even form networks with organizers to negotiate the details of upcoming protests before the organizers even submit a written notice. At this point, keeping communication channels with the protest organizers open and transparent is crucial.

##### 1.1.3.2. During a protest (Active)

To ensure peaceful protests, Korean dialogue police officers serve as a liaison not only between the police and protest organizers but also between the protesters and bystanders in the crowd (members of the general public affected by current rallies and marches) during the protest. Most importantly, they maintain rapport and mutual understanding by continuing the constructive dialogue and negotiation with the protest organizers in the crowd or nearby. In order to build rapport effectively, Korean dialogue police officers act in a humanitarian manner, such as caring for sick protesters or offering water bottles to some (weak) protestors during the hot and humid summer.

Korean dialogue police officers must separate potential agitators or aggressive protesters in the crowd from peaceful protestors and approach them individually to deal with them while closely monitoring the outcomes of self-policing. The Dialogue police officers must also make an effort to notice and analyze even the smallest alterations in crowd atmosphere and dynamics. They can explain potential police actions to the protest organizers based on respectful dialogue and cooperation, which can help tone down or minimize unnecessary tension and distrust. Dialogue Police officers can gather observable information during an event that can assist their commanders in making better decisions for the best possible outcomes for everyone involved. Based on dynamic risk assessment, despite these efforts at dialogue and negotiation (‘legitimacy’), there may still be a very high risk of confrontation(s) or violence. Proactive micro-management of demonstrations or protests using models like command and control or strategic incapacitation may be used in these circumstances, with the use of force (‘power’) at a corresponding level reserved as a last resort.

##### 1.1.3.3. After a protest (Reactive)

After an incident, the Korean dialogue police officers’ responsibilities shift to ensuring protesters’ safety and safe return until the crowd disperses completely. Afterward, in order to get constructive feedback and define areas for improvement, dialogue officers hold hot and cold (formal/informal) debriefing sessions (or follow-up meetings) with both key protest organizers and their police colleagues (dialogue police officers and riot police officers). In order to be prepared for future protests or marches, it is still important to maintain some sort of communication with protest organizers (at least, lose contact) even after the protest is over.

Globally, these dedicated units for dialogue are based on the negotiated management theory of protest control methods and the ESIM theory of crowd psychology. Therefore, they assume that the crowd psychology of the protest is not initially unified and consists of sub-groups with diverse social identities. Thus, the purpose of these dedicated units is the mitigation of violence or management of conflicts through dialogue and negotiation, thereby preventing the crowd psychology from developing into a unified identity that fights against the police.

Another objective of a dedicated unit (other than strengthening legitimacy) is to stay close to or within the crowd, appropriately sense changes in crowd psychology, and alert the riot police of the change. In case the dialogue police on-site determine that the crowd psychology of the protest is developing into a unified identity due to external stimuli, the minority that instigates illegal violence (riot) and the majority that only expresses their opinion (without the intent of rioting) must be separated. This allows the riot police to execute a differentiated response exclusively against the former group of protesters.

## Policy implications and conclusions

In terms of protest response strategies, legitimacy is directed toward the proactive prevention model, whereas power is directed toward the reactive response model based on physical force. Therefore, procedural justice must be guaranteed to ensure legitimacy. Nonetheless, when citizens and police have different perceptions of legitimacy or when the gap between these perceptions is wide, it is necessary that the police actively communicate, for example, by adopting a dialogic approach, since ensuring power holder legitimacy is also essential.

The above dialogic approach is actively utilized not only in Korea but also in the UK and Sweden. In practice, they have established and are operating dedicated units for dialogue, such as Dialogue police and/or (similar) PLO or Dialog Polis to ensure procedural justice. Regarding legitimacy, the dedicated units associated with the dialog police are strictly separated from the riot police (that manage Power), as demonstrated in the specific protocols. These units are based on the negotiated management theory as a protest control method and the ESIM theory as a crowd psychology theory.

Based on the theoretical background and the specific protocols of the European countries, the failure of agencies to respond appropriately and the fact that the recommendations for improvement are solely from a perspective of power are the two most problematic aspects of the US Senate examination report on the US Capitol riot. In this regard, it can be understood that the US Senate and some US law enforcement agencies view crowds on the basis of the classic crowd psychology theory, which posits that due to the anonymity of the crowd, individuals commit illegality and use violence more easily, and that such illegality is contagious, quickly spreading to the entire crowd. However, such response strategies based on power pose an inherent risk of triggering a vicious cycle of violence and confrontations between the police and the protesters, should BLM-like protests occur again in the future.

In conclusion, the US Senate Report focuses solely on the response after the protest has degenerated into a riot. In order to respond to such riots, it only considers power to identify problems and make recommendations for improvements. In other words, it can be assumed that the US Senate report perceives protests as a threat as opposed to a constitutional right that must be protected. As a result, the focus is on reactive responses to such threats. As explained above, legitimacy is a key mechanism through which protests degenerate into riots. To conclude, the US urban police, including the USCP, should actively consider implementing a protest response policy, such as the dialogic approach, to strengthen not only their power but also their legitimacy in responding to changes in crowd psychology.

## Data availability statement

The original contributions presented in the study are included in the article/supplementary material, further inquiries can be directed to the corresponding author.

## Author contributions

HK: conceptualization, methodology, and writing—original draft preparation. JL: formal analysis, writing—review and editing, and funding acquisition. All authors have read and agreed to the published version of the manuscript.

## Funding

This study was supported by the BK21 FOUR program (Education and Research Center for Securing Cyber-Physical Space) through the National Research Foundation (NRF) funded by the Ministry of Education of Korea (5199990314137).

## Conflict of interest

The authors declare that the research was conducted in the absence of any commercial or financial relationships that could be construed as a potential conflict of interest.

## Publisher’s note

All claims expressed in this article are solely those of the authors and do not necessarily represent those of their affiliated organizations, or those of the publisher, the editors and the reviewers. Any product that may be evaluated in this article, or claim that may be made by its manufacturer, is not guaranteed or endorsed by the publisher.
